# Weight loss is associated with plasma free amino acid alterations in subjects with metabolic syndrome

**DOI:** 10.1038/nutd.2016.5

**Published:** 2016-02-29

**Authors:** O Tochikubo, H Nakamura, H Jinzu, K Nagao, H Yoshida, N Kageyama, H Miyano

**Affiliations:** 1 Department of Occupational Health, Kanagawa Health Service Association, Yokohama, Japan; 2 Institute for Innovation, Ajinomoto Co., Inc., Kawasaki, Japan

## Abstract

**Objectives:**

The prevalence of metabolic syndrome is increasing worldwide, especially in Asian populations. Early detection and effective intervention are vital. Plasma free amino acid profile is a potential biomarker for the early detection for lifestyle-related diseases. However, little is known about whether the altered plasma free amino acid profiles in subjects with metabolic syndrome are related to the effectiveness of dietary and exercise interventions.

**Methods:**

Eighty-five Japanese subjects who fulfilled the Japanese diagnostic criteria for metabolic syndrome were enrolled in a 3-month diet and exercise intervention. The plasma free amino acid concentrations and metabolic variables were measured, and the relationships between plasma free amino acid profiles, metabolic variables and the extent of body weight reduction were investigated. Those who lost more than 3% of body weight were compared with those who lost less than 3%.

**Results:**

Baseline levels of most amino acids in the subset that went on to lose <3% body weight were markedly lower compared with the counterpart, although both groups showed similar proportional pattern of plasma amino acid profiles. The weight loss induced by the diet and exercise intervention normalized plasma free amino acid profiles. For those with a high degree of weight loss, those changes were also associated with improvement in blood pressure, triglyceride and hemoglobin A1c levels.

**Conclusions:**

These data suggest that among Japanese adults meeting the criteria for metabolic syndrome, baseline plasma free amino acid profiles may differ in ways that predict who will be more vs less beneficially responsive to a standard diet and exercise program. Plasma free amino acid profiles may also be useful as markers for monitoring the risks of developing lifestyle-related diseases and measuring improvement in physiological states.

## Introduction

Metabolic syndrome is a pathophysiological state that brings about diabetes, hypertension and dyslipidemia, and is also a major risk factor for cardiovascular disease or ischemic heart disease.^1, 2^ Metabolic syndrome is caused by the accumulation of visceral fat, which promotes insulin resistance and a decrease in adiponectin levels.^3^ Owing to the global shift toward a Western lifestyle of eating and sedentary habits, the prevalence of metabolic syndrome has been consistently increasing worldwide, especially in Asian populations.^2^ For example, in Japan, a previous study showed that the percentage of Japanese people with metabolic syndrome was 16.7% and 7.4% for males and females, respectively,^4^ and metabolic syndrome is becoming a serious social medical issue.

It is widely accepted that early detection and early intervention are critical for preventing lifestyle-related diseases, including metabolic syndrome.^1^ Recently, plasma free amino acid profiling has been identified as a potential biomarker for early detection.^5, 6, 7, 8, 9^ Plasma free amino acid profiles are altered by visceral fat accumulation^8, 10^ and insulin resistance.^6, 8, 11^ Several cohort studies demonstrated that plasma free amino acid alterations can be used to predict the future development of diabetes and cardiovascular diseases—even after adjustments for commonly accepted risk factors.^5, 7, 8, 9, 12^ Among the plasma free amino acids, branched-chain amino acid (BCAA) levels are elevated in obese humans and animal models.^13, 14, 15^ This elevation is caused by insulin resistance which decreases the utilization of amino acids and uptake of BCAAs into muscles.^16, 17^ Other studies have shown that insulin resistance decreases the expression of adipose tissue BCAA-catabolizing enzymes leading to decreased BCAA metabolism in visceral adipose tissue.^16, 18, 19^ Other plasma free amino acid levels are also altered in people with high visceral obesity:^10^ glutamate (Glu), serine (Ser), proline (Pro), glycine (Gly), alanine (Ala), tyrosine (Tyr), phenylalanine (Phe) and tryptophan (Trp). It is believed that this alteration is caused by a combination of insulin resistance-induced accelerated protein break down in muscle and changes in the gluconeogenesis set point in liver.

Although these plasma free amino acid alterations are potential biomarkers of metabolic change, little is known about whether the altered plasma free amino acid profiles in subjects with metabolic syndrome are related to effectiveness of diet and exercise interventions. The primary goal of this research was to characterize the plasma free amino acid profiles of subjects with metabolic syndrome before and after they underwent weight loss intervention and to examine any associations between plasma free amino acid changes and changes in weight or metabolic risk factors. In this study, we set the cutoff point of weight loss at 3% loss from the initial body weight according to a previous study^20^ enrolling 3480 Japanese subjects with metabolic syndrome or obesity-related diseases. That study reported that the minimum weight reduction required for an improvement in obesity-related risk factors, including blood pressure, hyperlipidemia and hyperglycemia, was 3%. We aimed to delineate the differences between individuals who successfully reduced their body weight by more than 3%, and individuals who lost less than 3% of their body weight or gained weight.

## Methods

### Ethics statement

This study was conducted in accordance with the Declaration of Helsinki, and the protocol was approved by the Ethics Committee for Clinical Research at Yokohama City University and Kanagawa Health Service Association. All the participants gave written, informed consent for inclusion before they participated in the study. All the data were analyzed anonymously by labeling a number ID throughout the study. The study was registered in the University Hospital Medical Information Network Clinical Trials Registry (UMIN-CTR) UMIN000017188.

### Subjects

Eighty-five Japanese subjects (Table 1) who were referred to consult a metabolic syndrome clinic of the Kanagawa Health Service Association and fulfilled the Japanese diagnostic criteria of metabolic syndrome^21^ were enrolled. Diagnostic criteria for metabolic syndrome in Japan include waist circumference >85 cm for men or >90 cm for women, plus at least two of the following: high-density lipoprotein cholesterol (HDL-C)<40 mg dl^−1^ or triglyceride level of (TG)⩾150 mg dl^−1^; fasting plasma glucose (FPG)⩾110 mg dl^−1^; or blood pressure⩾130/85 mm Hg (Table 1). All participants were otherwise generally healthy and at least 20 years of age (51.1±1.2 years). Exclusion criteria included pregnancy, severe mental disorders and cancer.

### Protocol of the dietary and exercise intervention program

All subjects received a standardized specific counseling guidance in Japan.^22^ Participants visited the metabolic syndrome clinic monthly and each visit included a physical exam and both dietary and exercise counseling. Participants took blood test at least at baseline and again at 3 months.

Dietary counseling was conducted by dietitians and physicians. Basic tenets of the counseling included recommended intake of fish, fruit, seaweed and vegetable to ensure proper energy from healthy sources during the program. Dietary guidance was individualized for each subject on the basis of the baseline diet assessment by a Food Frequency Questionnaire Based on Food Groups. Exercise counseling was conducted by physicians, on the basis of the 2006 Guidelines for the Treatment of Obesity.

Subjects were instructed to record their body weight twice per day, morning and evening, on an available home or fitness center scale. Number of walking steps was monitored using a pedometer daily. Blood pressure was measured twice per day in the morning and evening. The subjects were instructed to record weight, walking and blood pressure on forms that they brought to each monthly clinic visit, and the physicians and dietitians provided guidance as necessary.

### Measurement of biochemical variables and plasma free amino acid concentrations

Fasting peripheral blood samples were taken and analyzed using automatic analyzers for all biochemical tests as noted below. The following reagents were used: Pureauto S (Daiichi Pure Chemicals Co, Ltd, Tokyo, Japan) for the measurement of TG and FPG; CHOLETEST (Daiichi Pure Chemicals Co, Ltd) for the measurement of HDL cholesterol; and PAPIDIA Auto hemoglobin A1c (HbA1c: Fujirebio Inc., Tokyo, Japan) for the measurement of HbA1c.

For amino acid analyses, blood samples were collected in tubes containing disodium ethylenediaminetetraacetate and were immediately placed on ice. The plasma was prepared by centrifugation at 3000 r.p.m. at 4 °C for 15 min and then stored at –80 °C until analysis. The plasma free amino acid concentrations were measured by high-performance liquid chromatography–electrospray ionization mass spectrometry following precolumn derivatization, as described previously.^23, 24, 25, 26, 27^ The following 20 amino acids were measured: alanine (Ala), arginine (Arg), asparagine (Asn), citrulline (Cit), glutamate (Glu), glutamine (Gln), glycine (Gly), histidine (His), isoleucine (Ile), leucine (Leu), lysine (Lys), methionine (Met), ornithine (Orn), phenylalanine (Phe), proline (Pro), serine (Ser), threonine (Thr), tryptophan (Trp), tyrosine (Tyr) and valine (Val).

### Statistical analyses

The statistical analyses except for a two-way repeated measures analysis of variance were performed using the JMP 9.0.0 program (SAS Institute Inc., Cary, NC, USA). Subjects were divided into two groups according to their body weight reduction, (i) individuals who successfully reduced their body weight by more than 3% (WLG) and (ii) individuals who lost less than 3% of their body weight or gained weight (N-WLG).

For all the variables, average and standard error were calculated. We calculated 'amino acid index for visceral fat' on the basis of the plasma free amino acid levels that correlate with the visceral fat deposition measured by computed tomography scanning. The amino acid index for visceral fat is a multivariable linear regression model used to show the relationships between the plasma free amino acid profiles and the visceral fat area; it consists of Ala, Asn, Gly, Trp, Tyr and Val.^8, 10, 28^ Levene's test was performed to assess the equality of variance. If Levene's test was not significant, a two-way repeated measures analysis of variance followed by Sidak's multiple comparisons test was performed for the comparison of the variables between different groups, or between baseline and after the program using GraphPad Prism 6 (GraphPad Software, La Jolla, CA, USA). Statistical significance was set at *P*<0.05. If Levene's test was significant, Welch analysis of variance followed by a nonparametric Steel–Dwass multiple-comparison test was applied.

To clarify the pattern differences in plasma free amino acid profiles at baseline between the WLG and the N-WLG, each plasma free amino acid level was normalized using gender-specific reference intervals,^26^ which was determined using a total of 1890 individuals, and plotted.

For obtaining the cluster relationship among plasma free amino acid levels and metabolic variables, Ward's method was used to create a dendrogram.^29^ The clusters composed of changes in the degree of body weight reduction and changes in other variables, including plasma free amino acids, were obtained. This figure was drawn by 48 participants' data (WLG: 11 males, 13 females and N-WLG: 16 males, 8 females) who did not have missing data regarding all the variables used to draw.

## Results

### WLG and non-WLGs had different plasma free amino acid concentrations at baseline

**Figure 1:**
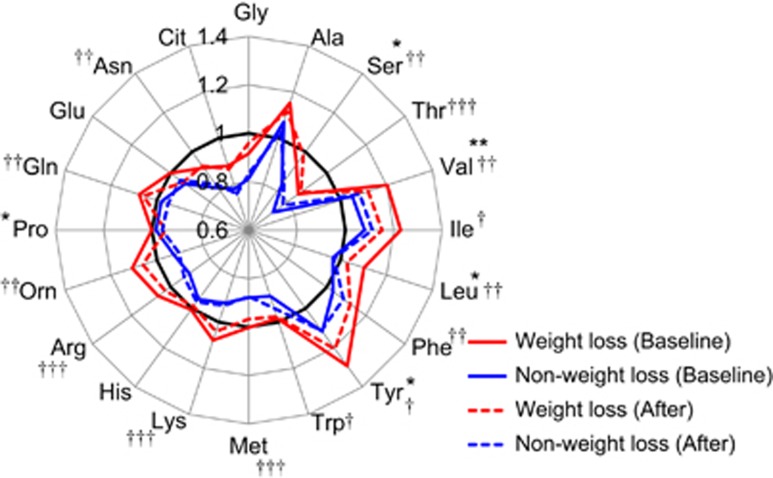
Plasma free amino acid profiles in the WLG and N-WLG. The plasma free amino acid level was normalized by healthy gender-specific reference intervals, and the average value of each group is plotted. The axes show the relative concentration of plasma free amino acids. Red lines indicate the WLG (*N*=50) and blue lines indicate the N-WLG (*N*=35). A two-way repeated measures analysis of variance followed by Sidak's multiple comparisons test was applied. Significant differences for comparisons between the baseline and after the program in WLG are shown as *P<0.05, **P<0.01. Significant differences between the WLG and N-WLG at baseline are shown as ^†^*P*<0.05, ^††^*P*<0.01, ^†††^*P*<0.001.

The changes in anthropometric and metabolic variables during the 3-month dietary and exercise intervention are shown in Table 1, separately for the weight loss group (WLG) vs non-weight loss group (N-WLG). Among the total 85 subjects, 50 subjects (27 males and 23 females) successfully reduced their body weight by more than 3% (WLG) and 35 subjects (26 males and 9 females) lost less than 3% of weight or gained weight (N-WLG). Accordingly, WLG lost 5.3±0.5 kg (average±s.e.; ranging from 22.0 to 2.0 kg), while N-WLG lost 0.8±0.2 kg (average±s.e.; ranging from 1.0 to −2.0 kg) after the intervention. After stratifying by weight loss success, we observed that the WLG and N-WLG differed significantly in their baseline levels of most amino acids (Figure 1), whereas very few differences were observed in other biochemical variables (Table 1). To clarify the pattern differences in plasma free amino acid profiles at baseline between the WLG and the N-WLG, each plasma free amino acid level was normalized using gender-specific reference intervals and plotted in Figure 1. From the figure, it is clear that the group exhibiting the WLG differed in baseline amino acid status as compared with N-WLG, specifically in that the WLG had a higher level of nearly every plasma free amino acid (all essential amino acids except for His and Arg, Orn, Gln, Asn and Ser) prior to initiating the diet and exercise program. Although nearly every plasma free amino acid level in baseline was higher in WLG than in N-WLG, the plasma amino acid proportional pattern was similar in both groups.

### Weight loss caused by dietary and exercise interventions improved metabolic variables

The baseline characteristics show a high percentage of subjects who were overweight (body mass index (BMI)⩾25 kg m^−2^; 91% of total subjects), hypertensive (systolic blood pressure⩾130 mm Hg; 66%, diastolic blood pressure⩾85 mm Hg; 59%), hyperglycemic (FPG⩾110 mg dl^−1^; 34%) and dyslipidemic (HDL cholesterol<40 mg dl^−1^; 9.5%, TG⩾150 mg dl^−1^; 42%); these values were based on the diagnostic criteria for metabolic syndrome in Japan. The WLG subset had a significant decrease in waist circumference and improvements in metabolic variables, including blood pressure, TGs (men only) and HbA1c after the program. On the other hand, as for the N-WLG subset, no significant improvement in metabolic variables was observed.

### The plasma free amino acid profiles were normalized by body weight loss

The normalization of circulating BCAA, Tyr and Pro levels (decreased), and Ser levels (increased) in the WLG after the 3-month program are depicted in Figure 1. However, because the proportion of males and females were largely different between the WLG and N-WLG, the plasma free amino acid changes were compared stratifying by gender (Table 2). In WLG, men had a significant decrease in BCAA and Pro levels. A similar, non-significant trend was seen in women. In the N-WLG men, concentrations of Thr increased significantly. For women in the WLG, the plasma free amino acid that changed significantly was Gly. To evaluate the overall trend in the plasma free amino acid profile changes, we investigated the change in the multivariable amino acid index, which is correlated with the amount of visceral fat. Significant reductions in the amino acid index for visceral fat were observed only in the WLG (Table 2).

**Figure 2:**
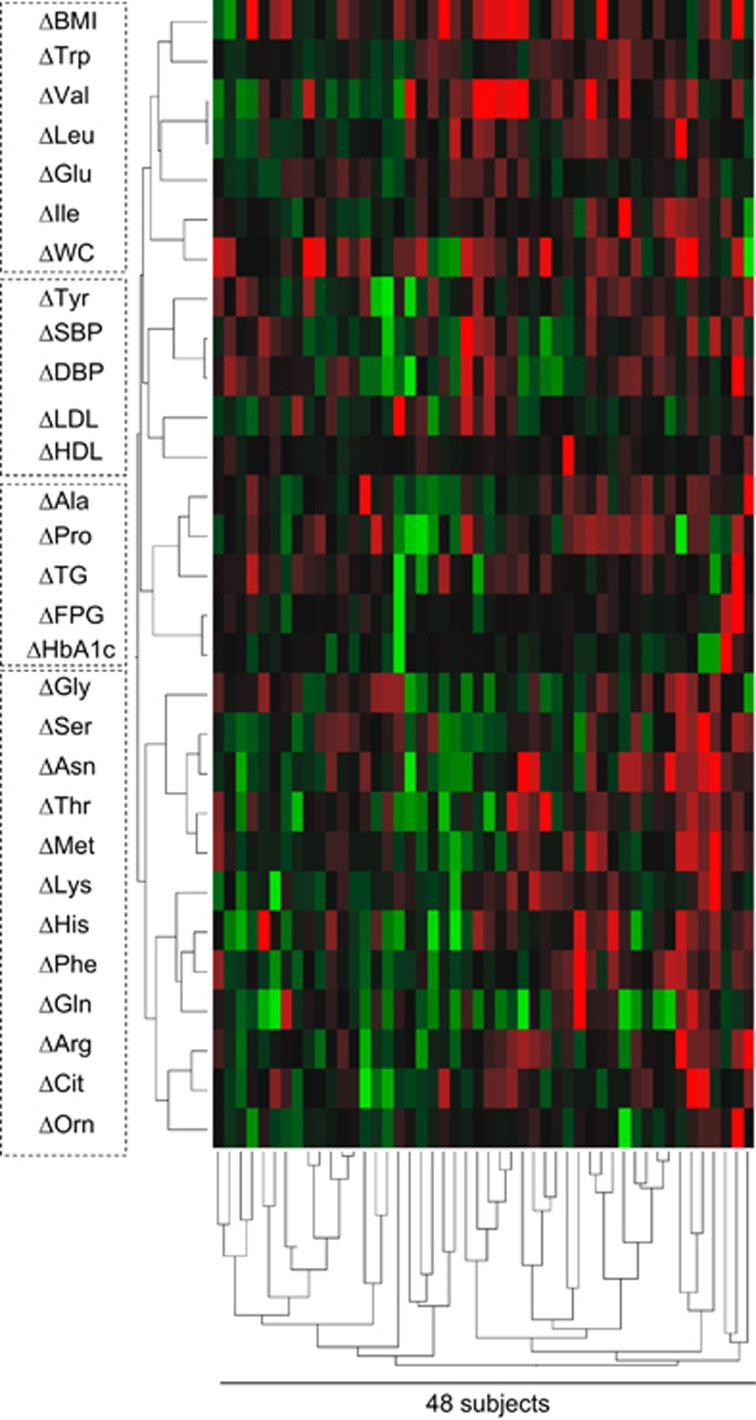
Cluster analysis of changing patterns of plasma free amino acid levels and other metabolic variables. The dendrogram was obtained by the hierarchical cluster analysis on the basis of the relative value of the difference (Δ) in each variable between baseline and after the program. The data from 48 participants (WLG: 11 males, 13 females and N-WLG: 16 males, 8 females) who did not have missing data were analyzed by Ward's method. The colored blocks represent the changes in the plasma free amino acid concentrations and metabolic variables of each subject. The green blocks represent relatively higher negative changes (higher reduction during the program), and the red blocks represent relatively higher positive changes (increase or smaller reduction during the program). The dotted line represents the cluster branches of the dendrogram.

The relationship between the normalization of plasma free amino acid profiles and improvement in metabolic variables is shown in Figure 2. This figure is drawn by 48 participants' data including the WLG and N-WLG. Cluster analysis was performed on the basis of the relative value of the difference (Δ) in each variable between baseline and post intervention. The colored blocks represent changes in the plasma free amino acid concentrations and metabolic variables for each subject. The green blocks show greater change in the negative direction (for example, higher reduction) from the beginning to the post program, and the red blocks show change in the positive direction (for example, a smaller reduction or even an increase). Figure 2 shows distinguishable clusters between plasma free amino acids and metabolic variables: for example, ΔVal, ΔLeu, ΔIle, ΔGlu and ΔTrp were in the same cluster as ΔBMI and Δwaist circumference. This indicates that these amino acids were altered along with changes in BMI and waist circumference. Similarly, ΔTyr was in the same cluster as ΔSBP, ΔDBP, ΔLDL and ΔHDL. ΔAla and ΔPro were in the cluster containing ΔFPG, ΔHbA1c and ΔTG.

## Discussion

To our knowledge, this study is the first to describe that initial plasma free amino acid profile may help to identify high vs low responders to a standardized diet and exercise weight loss program. In the current study, we demonstrated that the plasma free amino acid profiles are associated with subsequent weight loss success during a weight loss program. Moreover, the loss of body weight via dietary and exercise interventions normalized plasma free amino acid concentrations and there was a tendency for those with the greatest weight change to show the biggest change in amino acid levels. In addition, these changes were associated with improvement in blood pressure, TG and HbA1c levels.

An unexpected—but intriguing—finding in this study was the difference in the baseline condition of plasma free amino acid levels between the WLG and the N-WLG. Although the WLG and N-WLG were stratified at the end of the program according to the amount of weight loss, we observed that baseline plasma free amino acid concentrations of the subjects in the N-WLG (especially men) were significantly lower than those of the WLG (Figure 1 and Table 2); however, no clear differences were found in other metabolic variables (Table 1).

Alternative explanations for the baseline amino acid profile differences between WLG and N-WLG could involve differences in dietary habits or metabolic differences. One possibility is that low levels of circulating essential amino acids could hinder the amelioration of the metabolic syndrome condition. It was previously reported that plasma concentrations of amino acids become low and the balance of plasma free amino acid will be altered from control subjects when there is insufficient protein intake.^30, 31^ In addition, ingestion of whey protein or supplementation with essential amino acids help protein synthesis in the body and are known to aid in weight loss and prevent rebound weight gain after undergoing weight reduction.^32, 33^ Taken together, the failure to lose weight in the N-WLG may be due to insufficient intake of protein or essential amino acids, leading to the alteration of plasma free amino acid concentrations. To further elucidate this hypothesis, a prospective study comparing the dietary records of those in the WLG and the N-WLG is needed to investigate the effect of protein intake on weight loss and plasma free amino acid concentration. In such a study, it may also be important to examine how the effect might differ based upon sources of protein (for example, animal-based, fish-based or plant-based proteins). A final possibility is that metabolic and body composition differences in N-WLG could affect amino acid levels in the blood—specifically it has been reported that insulin and adiponectin levels cause fluctuation of blood amino acid levels.^6, 8, 11, 28^ Insulin and adiponectin also affect metabolism in liver, muscle and adipose tissues and dysregulation may hinder metabolic improvement. In this context, WLG subjects might be more 'naïve' to weight reduction due to the difference in their body composition. Further studies to investigate the amount of visceral fat and subcutaneous fat by computed tomography scanning could provide further information.

Several intervention studies have previously reported the normalization of plasma free amino acid profiles in morbidly obese patients.^14, 34, 35, 36^ One study showed that weight loss by Roux-en-Y gastric bypass surgery caused a decrease in plasma BCAA levels and that this decrease was associated with skeletal muscle insulin sensitivity.^34^ In a different study, the reduction in circulating levels of BCAA caused by Roux-en-Y gastric bypass was compared with the change caused by gastric banding vs a very low-calorie diet in morbidly obese patients; it demonstrated that Roux-en-Y gastric bypass had a larger effect on BCAA normalization.^35, 36^ However, the average BMI of patients who received surgical interventions was approximately 45 kg m^−2^, which is much higher than that of the individuals in this current study. Thus, the present study is the first to describe the plasma free amino acid normalization in patients with metabolic syndrome at lower BMI. One limitation of the study is that the higher amino acid levels at baseline might contribute to the normalization of their concentrations (mostly decreased after the program) in the WLG. Further confirmation by following the basal amino acid level matched-subjects will be needed.

Plasma free amino acid normalization was associated with body weight loss (Figure 1) and the improvement in metabolic variables (Figure 2). The improvement in metabolic variables in the WLG included decreases in waist circumference, blood pressure, TG level and HbA1c level (Table 1). The N-WLG showed no improved metabolic changes. BCAA, Ala, Pro, Tyr and Gly are known to have specific patterns in patients with lifestyle-related disease conditions including visceral obesity, insulin resistance, metabolic syndrome and type 2 diabetes.^8, 10, 28^ Our study supports this by showing a reversal of disease patterns, particularly in the WLG (Figure 1). Furthermore, the cluster analysis results in Figure 2 show that ΔTyr, ΔPro and ΔAla belonged to the cluster of Δ(blood pressure), Δ(lipid level), Δ(blood glucose level) and that ΔVal, ΔLeu, ΔIle, ΔGlu and ΔTrp were clustered with ΔBMI and Δwaist circumference. These findings suggest that the improvement in plasma free amino acid profiles may be related to body weight reduction and improved physiology. However, it is still possible that other factors including dietary habits (compliance to dietary guidance, type of diet), physical activities, medications and genetic background could affect the plasma amino acid profiles, and thus, further studies are wanted.

In conclusion, our study of 85 Japanese subjects with metabolic syndrome suggests that plasma free amino acid profiles normalized in association with weight loss and improvement in metabolic variables. Larger-scale studies are required to determine whether plasma free amino acid profiles could be used to evaluate patients for predisposition to effectiveness of weight loss programs.

## Figures and Tables

**Table 1:** Metabolic variables at baseline and post dietary and exercise intervention

		*Weight loss (WLG)*			*Non-weight loss (N-WLG)*		
		*Male* N=*27 Female* N=*23*			*Male* N=*26 Female* N=*9*		
Age (years)	*M*	49.6±2.4			50.7±2.1		
	*F*	52.6±1.9			54.2±4.6		
		*Baseline*	*After*	P	*Baseline*	*After*	P
Height (cm)	M	170.8±0.9			169.8±1.5		
	F	156.9±1.5			156.7±1.8		
Weight (kg)	M	**84.3±2.5**	**77.8±2.2**	***	81.9±2.5	81.1±2.6	
	F	**71.6±2.1**	**67.8±1.9**	***	72.2±3.9	71.2±3.9	
Body mass index (kg m^−2^)	M	**28.8±0.7**	**26.6±0.7**	***	28.3±0.7	28.1±0.8	
	F	**29.1±0.8**	**27.5±0.7**	***	29.8±2.0	29.4±2.0	
Waist circumference (cm)	M	**100.2±2.5**	**95.1±2.3**	***	96.2±2.0	95.3±2.1	
	F	**96.1±1.9**	**93.2±1.7**	***	102.6±3.7^†^	101.7±3.9	
SBP (mm Hg)	M	**139.9±3.9**	**126.5±4.6**	***	137.4±3.7	135.0±4.2	
	F	**140.9±4.0**	**131.4±3.0**	**	138.1±5.5	133.9±4.2	
DBP (mm Hg)	M	**90.5±3.0**	**82.3±2.7**	**	85.5±2.5	85.9±3.3	
	F	**87.7±2.2**	**80.7±2.4**	**	87.1±4.5	80.8±3.1	
HDL cholesterol (mg dl^−1^)	M	59.3±8.6	52.7±2.2		56.2±2.8	60.6±3.3	
	F	55.8±2.1	59.7±3.5		62.7±6.0	62.1±3.9	
Triglyceride (mg dl^−1^)	M	**190.5**±**36.9**	**130.8**±**22.7**	*	183.7±29.3	158.6±31.2	
	F	116.2±8.0	129.6±9.7		170.8±32.5	121.7±17.0	
Fasting plasma glucose	M	114.1±7.9	104.8±3.8		103.6±4.9	115.1±8.6	
(mg dl^−1^)	F	97.4±4.0	97.2±1.7		112.4±6.2	107.4±5.6	
HbA1c (%)	M	**5.9**±**0.3**	**5.2**±**0.1**	*	5.6±0.2	5.8±0.5	
	F	**5.6**±**0.1**	**5.3**±**0.1**	**	5.9±0.4	5.8±0.3	

Abbreviations: DBP, diastolic blood pressure; F, female; HDL, high-density lipoprotein; M, male; N-WLG, non-weight loss group; SBP, systolic blood pressure; WLG, weight loss group. The body weight decreased by more than 3% in the WLG, whereas the N-WLG lost less than 3% of the body weight at the end of the program. A two-way repeated measures analysis of variance followed by Sidak's multiple comparisons test was applied. Significant differences for comparisons between the baseline and after the program are shown as *P<0.05, **P<0.01, ***P<0.001 and are highlighted in bold letters. Significant differences between the WLG and N-WLG at baseline are shown as ^†^*P*<0.05. The data are expressed as the average±s.e.

**Table 2:** Plasma free amino acid profiles at baseline and post dietary and exercise intervention

		*Weight loss (WLG) (*N=*50)*			*Non-weight loss (N-WLG) (*N=*35)*		
		*Baseline*	*After*	P	*Baseline*	*After*	P
*Essential amino acids* (μmol l^−1^)							
Val	M	**264.4**±**7.7**	**243.8**±**8.0**	*	231.8±7.2^††^	241.2±8.5	
	F	249.5±9.1	234.5±7.0		243.2±8.3	245.2±14.3	
Ile	M	**76.7**±**2.9**	**69.2**±**2.2**	*	66.7±3.0^†^	70.6±3.0	
	F	68.2±3.1	66.2±2.6		69.9±6.4	68.0±5.4	
Leu	M	**137.3**±**3.7**	**123.2**±**3.6**	*	119.4±5.3^††^	121.7±4.0	
	F	118.2±4.1	114.8±4.0		117.7±6.2	114.1±8.3	
His	M	86.2±2.5	81.5±2.0		79.1±2.6	79.6±1.9	
	F	76.1±1.6	78.4±1.6		76.6±4.3	79.2±3.3	
Phe	M	67.6±2.3	65.7±1.7		61.0±1.5^†^	65.2±1.8	
	F	65.0±2.2	62.9±2.0		60.1±2.1	60.3±2.9	
Trp	M	61.0±1.8	58.5±1.7		55.1±2.0	58.2±1.9	
	F	53.7±2.3	55.3±2.0		48.3±2.9	51.6±2.7	
Met	M	26.9±1.0	25.5±0.8		23.4±1.0^†^	23.6±0.8	
	F	23.7±0.6	23.4±0.6		21.7±0.6	20.8±1.0	
Thr	M	110.2±3.7	110.0±3.4		**90.9**±**4.6**^††^	**99.2±4.2**	*
	F	100.7±4.4	104.5±4.5		92.8±6.4	93.1±4.6	
Lys	M	200.5±7.1	193.9±5.1		176.7±5.0	176.3±4.4	
	F	191.9±5.0	184.5±4.6		159.6±6.7^††^	167.4±11.7	
*Non-essential amino acids* (μmol l^−1^)							
Ser	M	110.8±4.5	117.3±4.3		98.7±3.0	100.4±3.1	
	F	108.3±3.5	112.8±3.9		92.5±4.0^†^	94.4±5.3	
Gly	M	216.7±9.5	221.3±7.9		189.2±7.1^†^	194.7±6.1	
	F	**233.0**±**12.1**	**252.1**±**14.0**	**	196.6±24.1	197.4±23.3	
Ala	M	404.4±17.7	366.2±8.3		368.0±16.9	367.1±14.0	
	F	389.6±18.9	402.9±17.4		403.0±18.5	355.9±18.7	
Tyr	M	78.8±3.9	73.1±2.8		67.3±3.3^†^	71.5±2.9	
	F	79.9±4.3	75.1±3.8		**77.9**±**6.3**	**67.1**±**4.2**	*
Glu	M	43.9±3.3	38.4±2.6		43.9±3.6	43.2±3.2	
	F	38.0±4.3	37.8±2.9		32.7±3.5	37.6±6.3	
Pro	M	**154.4**±**7.0**	**142.5**±**6.8**	*	158.7±12.1	157.7±11.8	
	F	142.3±8.5	137.3±8.4		143.1±10.2	132.7±11.0	
Gln	M	607.8±16.3	599.5±15.3		560.2±12.1^†^	541.0±12.0	
	F	606.1±14.1	593.4±19.2		566.0±21.8	556.6±33.4	
Cit	M	27.7±2.3	26.0±2.0		23.2±2.1	22.9±1.7	
	F	26.7±1.9	27.7±1.9		28.2±3.2	26.4±3.2	
Arg	M	99.6±4.1	94.0±3.1		81.9±3.0^†††^	85.8±3.1	
	F	89.8±3.8	89.2±2.5		83.3±4.7	81.9±8.1	
Orn	M	56.1±2.6	55.5±2.2		47.7±1.9	46.7±4.1	
	F	57.1±2.5	53.0±2.2		52.7±4.4	50.9±5.6	
Asn	M	45.4±1.6	43.9±1.2		37.6±1.4^†††^	39.7±0.9	
	F	40.3±1.2	43.1±1.4		41.1±2.2	39.0±2.8	
*Multivariable amino acid index*							
Amino acid index for visceral fat	M	**15.8**±**0.9**	**13.8**±**0.8**	*	14.8±0.7	14.9±0.7	
	F	**16.1**±**1.1**	**14.0**±**1.0**	**	17.1±0.8	15.0±1.5	

Abbreviations: F, female; M, male; N-WLG, non-weight loss group; WLG, weight loss group. The body weight decreased by more than 3% in the WLG, whereas the N-WLG lost less than 3% of the body weight at the end of the program. Amino acid indices are based on the multivariable regression model for modeling the relationships between plasma free amino acid profiles and visceral fat accumulation. A two-way repeated measures analysis of variance followed by Sidak's multiple comparisons test was applied. Significant differences for comparisons between the baseline and after the program are shown as *P<0.05, **P<0.01 and are highlighted in bold letters. Significant differences between the WLG and N-WLG at baseline are shown as ^†^*P*<0.05, ^††^*P*<0.01 and ^†††^*P*<0.001. The data in the tables are expressed as the average±s.e.
